# The Effect of rs80860411 Polymorphism on Fattening, Slaughter, and Pork Quality Traits in Polish Large White and Pulawska Breeds

**DOI:** 10.3390/ani15142090

**Published:** 2025-07-15

**Authors:** Anna Antonyk, Arkadiusz Terman, Mirosław Tyra, Grzegorz Żak, Daniel Polasik, Magdalena Szyndler-Nędza, Hanna Kulig, Andrzej Dybus

**Affiliations:** 1 Department of Genetics, West Pomeranian University of Technology in Szczecin, 70-311 Szczecin, Poland; anna.gluszyk@zut.edu.pl (A.A.); 2 Department of Pig Breeding, National Research Institute of Animal Production, 32-083 Balice, Poland; miroslaw.tyra@iz.edu.pl (M.T.); grzegorz.zak@iz.edu.pl (G.Ż.)

**Keywords:** rs80860411, *GALNT15*, fattening traits, slaughter traits, meat quality traits, pigs

## Abstract

The intergenic SNP (single-nucleotide polymorphism) rs80860411A>C was identified as a major QTL for drip loss measured on semimembranosus muscle (SM) in pigs. The SNP is located near the *GALNT15*. The purpose of this study was to analyze the association between rs80860411A>C and fattening, slaughter, and quality traits of Polish pigs. This study was conducted on 235 individuals belonging to two breeds, Polish Large White (n = 187) and Pulawska (n = 48). The rs80860411 genotypes were determined using the PCR-RFLP method. Association analysis was performed for each breed separately. It was shown that rs80860411A>C had a significant effect on fattening performance traits, on several slaughter performance traits, including width of the loin eye and carcass meat content (*p* ≤ 0.01, *p* ≤ 0.05), as well as on meat color—redness (a*) (*p* ≤ 0,05) in Pulawska breed. The obtained results indicate that the studied SNP has the potential to be a QTN and could be included in pig selection programs, especially in Pulawska pigs.

## Simple Summary

The aim of this study was to analyze the association between intergenic SNP rs80860411A>C and fattening, slaughter, and pork quality traits of pigs. The association studies included a total of 235 individuals belonging to the Polish Large White and Pulawska breeds. It was found that rs80860411A>C has a significant effect on fattening performance traits, as well as on several slaughter performance traits: width of the loin eye, loin and ham weights without skin and backfat, and carcass meat content in the Pulawska breed. The studied SNP was found to affect such quality traits as meat color—redness (a*) in the Pulawska breed and loin texture in Polish Large White. So, the studied SNP could be useful in pig selection programs for improving the fattening, slaughter traits, and meat quality traits, in particular in the Pulawska breed

## 1. Introduction

The situation in pig farming in Poland should be considered unfavorable. The total pig population at the end of 2024 was about 9078.3 million, and compared to the previous year, it fell by 7.1% [1]. The situation of declining herds has also been observed for many years with regard to pedigree breeding. Over the past 10 years, the total number of evaluated pedigree sows fell by about 58.6%, the number of breeding gilts under evaluation fell by 80.1%, and young boars by 84.4% [2,3].

Such a situation has resulted in Poland, which was once a significant producer and exporter of pork, becoming an importer for several years. A number of factors have contributed to the unfavorable situation of the pig breeding and production sector in Poland. Among those that had the greatest impact were the appearance of ASF on Polish territory in 2014 and the associated restrictions, large fluctuations in the yields of cereals and feed crops used in pig feeding due to adverse climate changes, which in effect contributed to an increase in feed prices. Not insignificant were unstable and usually low, even below the cost of production, purchase prices of fattening pigs and breeding material, a significant increase in energy prices, which caused many farmers in Poland to give up pig breeding and production. In the European Union, the trend is similar to that in Poland. In December 2024, the EU pig population was 131.98 million, down 0.7% from 2023 [4].

Meat quality is a very important aspect of pig breeding. The definition of quality was created centuries ago, and the first mentions in the literature indicate the ancient period. This concept is not easy to define because it depends on many factors. In addition to genetic factors, such as breed and sex, and physiological factors (age, condition, health), meat quality is also influenced by environmental factors (animal husbandry system, nutrition, transport, slaughter, and post-slaughter processing). One of the most important economic characteristics of pigs is the weight of the most valuable carcass cuts and the growth rate of fattened pigs. Their development and growth are factors that ensure the profitability of breeding and an adequate supply of meat consumed by humans [5,6].

The basic features of meat quality, including technological and consumer suitability, include primarily color, pH, uniformity, and durability. Other important features include water binding and holding capacity, as well as emulating and gelling properties. In turn, texture, tenderness, juiciness, taste, and aroma are features that determine the appropriate meat quality as a raw material. The features that affect meat tenderness include high moisture content, intramuscular fat, and muscle fiber structure. Intramuscular fat and moisture content are positively correlated with meat texture, while muscle fiber diameter is inversely proportional to many quality features, including meat texture. Predominantly red fiber muscles have smaller fiber size, higher fiber density, enhanced tenderness, lower shear force, and more pronounced flavor characteristics [7,8,9].

The research results show that the color and pH of meat are closely related to its quality. They largely affect the freshness, shearing force, and drip loss of meat. The traits characterized by low heritability and the occurrence of differences in individual populations include pH and meat color (L*—lightness; a*—redness; b*—yellowness) [10,11,12].

One of the main goals from the breeding point of view is, among others, to establish a genetic marker that would allow the control of fatness in pigs without losing the already achieved level of meatiness.

Analysis of genes regulating skeletal muscle development is of great importance for understanding the molecular basis of this phenomenon. Polymorphic variants of many genes affect the differentiation of fattening efficiency. The results of genetic and genomic studies published in recent years have significantly contributed to a better understanding of many molecular mechanisms involved in the process of muscle tissue synthesis [13,14].

Genetic markers that are predictive of pork quality can be used in selection programs [15]. The recessive allele of the *RYR1* gene was considered for years to be the most important genetic marker in pig breeding [16]. Currently, polymorphism in intron 3 of the *IGF2* gene, encoding insulin-like growth factor 2, is the most important genetic marker for fattening, slaughter, and meat quality traits in pigs [17,18,19]. Thanks to the availability of genome editing techniques, the mutation in the *IGF2* gene has been introduced to the gene pool in breeds in which it does not occur naturally [20]. Other important genetic polymorphisms for pig breeding have been located in the *MC4R*, *CAST*, and *PRKAG3* genes [15,21].

An effective technique for identifying common DNA sequence variation, including genetic variants that affect various traits of farm animals, is WGS (Whole Genome Sequencing) [22]. Many SNPs have been described in the pig genome, which are located in both genic and intergenic regions [5]. It is worth mentioning that intergenic SNPs are considered important factors that participate in the regulation of gene expression [23].

Since genotype is one of the main factors determining the composition and quality of the carcass, research has been carried out for years to identify QTL (Quantitative Trait Loci) and QTN (Quantitative Trait Nucleotide) [24,25]. Thanks to the use of genome-wide association studies (GWAS), the identification of polymorphic variants with QTN potential has become feasible. The analysis of the occurrence of polymorphic variants (most often SNPs) is widely used to detect genotype-phenotype associations [26,27,28,29].

As a result of the study of the pig genome, a huge amount of information has been obtained, which has been helpful in gaining new knowledge about biological systems. This has opened up new possibilities for genetic selection in this species. Through the integrative analysis of GWAS, eQTL (Expression quantitative trait locus) and QTT (Quantitative trait transcript) loci, a major QTL for drip loss of SM in SSC13 was identified. It turned out to be the SNP rs80860411. QTT analysis showed that the nearby gene *GALNT15* was characterized by a negative correlation of expression level with the drip loss phenotype [29]. Hence, *GALNT15* was considered a strong candidate gene influencing drip loss.

It can be assumed that the rs80860411 intergenic SNP plays a role in regulating the activity of the *GALNT15* gene; therefore, the aim of this study was to analyze the association between the SNP and fattening, slaughter, and pork quality traits of Polish pigs.

## 2. Materials and Methods

### 2.1. Animals and Nutrition

The research was conducted using material from 235 individuals of two breeds, Polish Large White (n = 187) and the native Pulawska breed (n = 48). The Polish Large White animals came from 40 sires and 114 dams, and the Pulawska from 15 sires and 32 dams. The studied groups of animals were not significantly related to each other; therefore, the sire effect was not included in the statistical models.

The material was obtained from Pig Performance Control Stations located in Poland, where routine pig assessment is conducted. Therefore, there was no need for the approval of the research by the bioethics committee.

Animals were fed, maintained, and slaughtered under standard conditions to determine their fattening and slaughter performance and estimate their breeding value. The pigs were kept in individual pens at the time of the test proper, that is, from 30 to 100 kg of body weight. During fattening, they received two types of feed (from 30 to 80 kg body weight and from 80 to 100 kg body weight) fed ad libitum until they reached a body weight of 100 (±2.5) kg. Slaughter was carried out by bleeding after stunning with high-voltage electric forceps.

### 2.2. Fattening Performance Test

During the experiment, the following fattening traits were recorded at the experimental stations: average daily gain from 30 to 100 kg of body weight as test daily gain (g/day), lifetime daily gain (g/day), daily feed intake (kg), feed conversion ratio (kg/kg gain), age at slaughter (days) and number of days in the test (days) [30,31].

### 2.3. Carcass Traits

After slaughter, the half carcasses were cut into pieces, taking into account the parameters of muscle content, fat content, and basic meat quality features in accordance with the station methodology procedure. The following measurements were made: carcass yield (%), middle length of carcass (cm), loin weight (kg), loin and ham weights without skin and backfat (kg), width of loin eye (cm), height of loin eye (cm), loin eye area (cm^2^), carcass meat content (%), average backfat thickness (cm) and weight of primary cuts (kg).

The average backfat thickness was determined using five measurements, which included the thickest point over the shoulder blade, the place on the back above the joint located between the last thoracic vertebra and the first lumbar vertebra, and three measurements over the loin—above the rostral, middle and caudal edge of the gluteus muscle cross-section [32]. The loin eye area was determined by cutting the muscle between the last thoracic vertebra and the first lumbar vertebra. The measurement was made based on the contour made in the cephalic plane of the plane of cut.

### 2.4. Meat Quality Traits

Among the meat quality parameters, the intramuscular fat content (IMF), pH, color, water holding capacity (WHC), and texture parameters were assessed.

To assess IMF content, samples were taken from the middle cross-sectional area of the *M. longissimus lumborum*, cut behind the last rib. The muscle was homogenized, and the crude fat was extracted by the Soxhlet method using the Soxtherm SOX 406 system (Gerhardt, Königswinter, Germany) [33]. Meat pH was determined in the *M. longissimus lumborum* and *M. semimembranosus* 45 min (pH 45) and 24 h (pH 24) after slaughter using a pH-Star apparatus (Matthäus, Eckelsheim, Germany). The loin color parameters—lightness (L*), redness (a*), yellowness (b*)—were measured using a CR-310 Chroma Meter (Minolta Konica, Tokyo, Japan). WHC was determined as the free water content in raw meat according to the Grau–Hamm method (a filter paper between two glass tiles) [34].

Meat texture measurements were performed on the *M. longissimus lumborum* and *M. semimembranosus* using the Warner–Bratzler shear force (WBSF) and texture profile analysis (TPA) methods. The WBSF test was used to determine firmness and toughness. It was performed on raw and cooked meat using a TA.XTPlus texture analyzer (Stable Micro Systems, Godalming, UK). Texture profile analysis was used to determine hardness, springiness, cohesiveness, chewiness, and elasticity. TPA was performed using the same device equipped with a 50 mm diameter cylinder [35].

### 2.5. Genotyping

Genomic DNA was isolated from samples of the *longissimus dorsi* muscle. The following kits were used for this purpose: Genomic Mini and Sherlock AX (A&A Biotechnology, Gdansk, Poland) and ReliaPrep^TM^ gDNA Tissue Miniprep System (Promega, Madison, WI, USA).

Genotypes were analyzed by PCR-RFLP. PCR products were amplified using the following primers (Table 1) that were designed using the PRIMER3 program (https://primer3.ut.ee/, accessed on 11 July 2025).

The PCR mixture contained ~100 ng of genomic DNA, 12 pmol of each primer, 1× PCR buffer (with 2 mM MgCl_2_), 0.2 mM dNTP mix, 0.3 U *Taq* polymerase, and deionized water (up to 12 µL). The following thermal profile was used: 94 °C for 5 min, followed by 35 cycles of 94 °C for 30 s, 60 °C for 35 s, and 72 °C for 50 s and a final extension at 72 °C for 5 min. The obtained amplicons (842 bp) were digested with the restriction enzyme *Dra*I (EURx, Gdańsk, Poland) for 2–3 h at 37 °C. The resulting DNA fragments were separated electrophoretically in 2% agarose gels stained with ethidium bromide in 1× TBE buffer (120 V, 40 min). The results were visualized under UV light and archived (Quantum, VILBER, Collégien, France).

The existence of the *A/C* polymorphism was confirmed by sequencing (Sanger method) of homozygous genotypes (*AA* and *CC*) for the rs80860411. Chromas software (https://technelysium.com.au/wp/, accessed on 11 July 2025, version 2.6.6) was used to analyze the sequencing results.

### 2.6. Statistical Analysis

In each analyzed herd of pigs, the frequencies of genotypes and alleles were estimated. The frequencies of individual genotypes within breeds were compared using the chi-square test. 

In the analysis of the association between the tested genotype and fattening, slaughter performance traits, and meat quality traits, the following linear model (General Linear Model) was applied using SAS software (ver. 8.02):

Y_ijk_ = μ + b_i_ + g_j_ + (bg)_ij_ + βSW_k_ + e_ijk_

where:

Y_ijk_—observation;

μ—overall mean;

b_i_—fixed effect of i breed;

g_j_—fixed effect of j genotype;

(bg)_ij_—interaction between i breed and j genotype;

βSW_k_—linear effect of slaughter weight as covariate (for slaughter traits only);

e_ijk_—random residual error.

The least squares method (LSM) was used to determine the significance of differences between genotype groups.

## 3. Results

**Figure 1:**
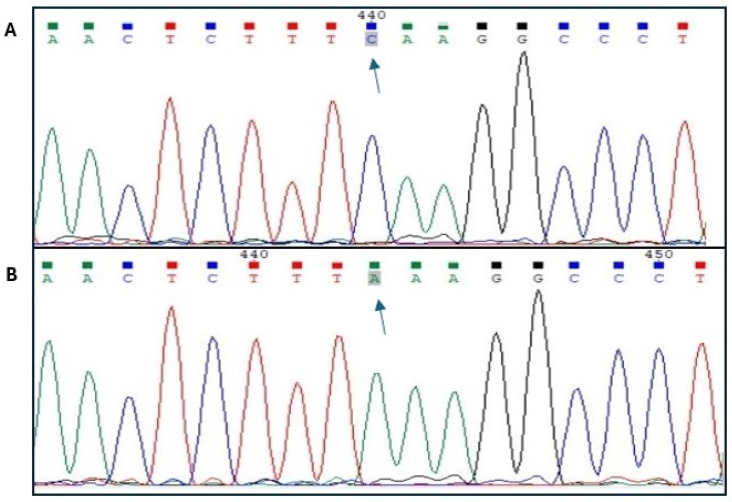
Sequencing results for the polymorphic site rs80860411A>C ((**A**)—genotype *CC*; (**B**)—genotype *AA*).

In order to confirm the occurrence of the rs80860411 polymorphism, sequencing of samples designated as homozygous genotypes was performed (Figure 1).

**Figure 2:**
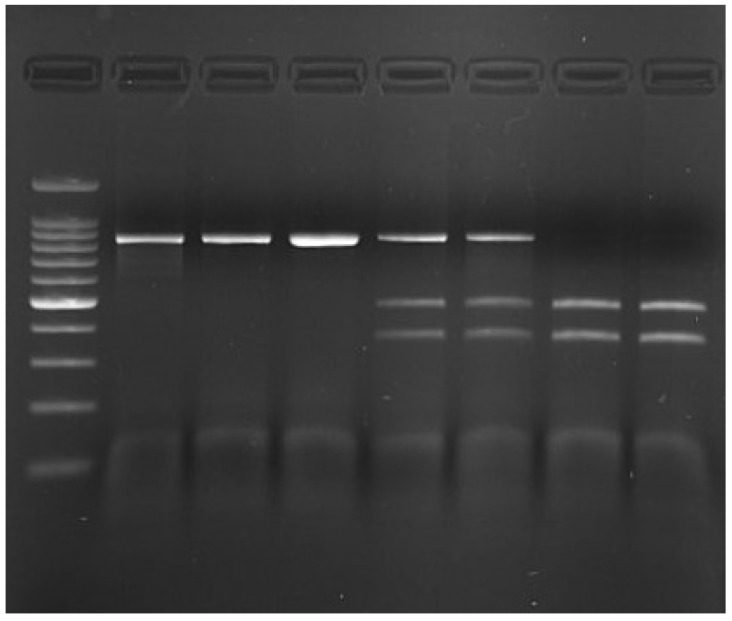
Results of rs80860411A>C genotyping by PCR-RFLP method; from left: lane 1—100 bp DNA Ladder (Promega; The 500 bp fragment is present at increased intensity); lanes 2, 3, 4—*CC* genotype, lanes 5 and 6—*AC* genotype, lanes 7 and 8—*AA* genotype.

The obtained PCR products of 842 bp length were digested with the *Dra*I enzyme. This allowed for the determination of three genotypes based on the obtained restriction fragments (Figure 2).

In this study, the highest frequency of the *AC* genotype was observed in the Pulawska breed, while in the Large White, the *CC* genotype was observed. In the two breeds studied, the *AA* genotype was found to be least frequent (Table 2).

The Hardy–Weinberg equilibrium was checked using the chi-square test. The observed and expected genotype frequencies in Pulawska and Polish Large White breeds did not differ significantly, so these herds were in Hardy–Weinberg equilibrium (Table 2).

The presented study showed significant associations between the rs80860411 polymorphism and fattening traits, as well as slaughter traits in the Polish Pulawska breed (Table 3 and Table 4).

A significant effect of the analyzed SNP was demonstrated on all fattening traits examined, except for daily feed intake, in the Pulawska breed. Individuals with the *AC* and CC genotype were characterized by a significantly higher test daily gain (*p* ≤ 0.01) and lifetime daily gain (*p* ≤ 0.05) compared to *AA* homozygous individuals. However, in pigs with the *AA* genotype, the highest number of days on test, feed conversion, and age at slaughter were observed; these traits were significantly higher (*p* ≤ 0.01, *p* ≤ 0.05) than in heterozygotes and *CC* homozygous pigs. In the case of the remaining two breeds, no significant differences were found between the values of the analyzed traits.

The statistical analysis of slaughter traits, taking into account the right half-carcass, showed highly significant relationships (*p* ≤ 0.01) between the rs80860411 polymorphism and the width of the loin eye, the carcass meat content, and the weight of primary cuts in the Pulawska breed. The values of these traits were significantly higher in individuals with the *AA* and *AC* genotypes compared to *CC* homozygotes. This polymorphism was also significantly associated (*p* ≤ 0.05) with the loin eye area in the Pulawska breed. In the case of Polish Large White pigs, no significant relationships were found between the analyzed polymorphism and the traits included in this study.

Referring to meat quality traits, statistically significant associations (*p* ≤ 0.05) were found between the rs80860411 polymorphism and meat color (redness, a*) in the Pulawska breed. The value of this trait was the highest in individuals with the *AA* genotype. In the case of Polish Large White, no effect of the studied SNP on the analyzed traits was found (Table 5).

In the case of loin texture, statistically significant associations were found between the rs80860411 polymorphism and firmness (r) in the Polish Large White breed (Table 6, *p* ≤ 0.05); and no associations when the day of slaughter was taken into account in the statistical model (Table S1).

In the case of ham texture, no statistically significant differences were found between genotypes and analyzed traits (Table S2). Individuals with different genotypes belonging to the Polish Large White breed differed significantly in firmness (r) and toughness (r) values only when the day of slaughter was included in the statistical model (Table S3). The values of these traits were significantly higher in individuals with the *CC* genotype in each case compared to individuals with the other genotypes.

## 4. Discussion

In the current literature, there is an increasing number of publications on gene searches for the detection of SNPs. Ahmed et al. [36] in their work recorded nearly 56.5 million SNPs in the genomic data of 155 cattle samples. About 25.87 million biallelic SNPs were identified in Kashmere cattle, occurring mainly in intergenic (58.20%) and intronic (37.71%) regions. Only 0.85% of all SNPs were detected in exons. Additionally, 0.9% of SNPs were found in untranslated regions and 0.1% in splicing sites. Similarly, in water Buffalo, the SNP localization by region was higher in the intronic region (61.69%), followed by the intergenic region (17.71%), than, for example, in exons (1.78%) [37]. Analysis of the pig genome also revealed many SNPs. In the genic region, 19,672 SNPs were described, with the largest number (15,824 SNPs) in introns. In the intergenic region, 20,055 SNPs were described [38]. Also in the human genome, the most significant variants have been identified in introns and intergenic regions [39].

Single-nucleotide polymorphism is considered one of the most important molecular markers. SNPs are widely used in animal breeding programs aimed at improving desired traits. Due to the wide distribution of intergenic SNPs in the pig genome, it is worth paying more attention to them [28,39].

The Polish Large White breed selected in this study is the main maternal breed for commercial crossbreeding. Importantly, they are also characterized by good meat parameters. The Pulawska breed is one of the oldest Polish pig breeds. The material consisted of crossbreeds of primitive local pigs with imported breeds, mainly Berkshire, and then refined with the Large White breed. Finally, the breed was recognized in 1935 under the name Golebska, and in 1951 it was registered as Pulawska. It represents a type between fat-meat and meat types. The characteristics of this breed include the following: longevity, resistance to diseases and less favorable environmental conditions, early maturation, and good maternal traits [40]. The breed is covered by a Genetic Resources Conservation Program, the most important assumptions of which are to increase the number of animals, maintain valuable breed features, maintain a constant level of genetic distinctiveness, and intra-breed variability. These tasks are supervised by the National Research Institute of Animal Production [41]. Pigs of this breed are usually kept on small family farms [42]. The number of Pulawska pigs changed in subsequent breeding periods depending on the preferences of the meat market. As the data show, the number of sows of this breed has a constant upward trend. In 2023, the number of sows covered by the breeding program was 2668 individuals [3]. For comparison, in 2013, it was only 590 individuals [2].

Fattening, slaughter, and meat quality traits are characterized by high phenotypic variability. The heritability coefficients for selected traits were estimated as follows: loin eye area (cm^2^)—0.418 [43], lifetime daily gain (g/day)—0.490 [43], IMF 0.50–0.62 [44,45], This indicates a significant contribution of genetic background in the formation of this group of traits.

Hence, it is indicated that they can be improved or modified using appropriate genetic markers [46]. In the presented study, significant associations were found between the rs80860411A>C genotypes and fattening performance traits, as well as several slaughter performance traits, including the width of the loin eye and carcass meat content (*p* ≤ 0.01, *p* ≤ 0.05) in Pulawska breed pigs. In the case of the Polish Large White, no influence of the polymorphism was found on the examined traits. The analysis of meat quality traits showed that the studied SNP had a significant effect on meat color—redness (a*) in the Pulawska breed. In the Polish Large White pigs, significant differences were also found for firmness (r) in individuals with different genotypes.

Previous studies have shown that the meat of Pulawska pigs was characterized by a significantly darker color and lower water content compared to other breeds [7,47]. Raw meat of Pulawska pigs has been found to have a higher protein and mineral content, which indicates a higher nutritional value compared to the meat of hybrid pigs [7]. A consumer study by Channon et al. [48] showed that more red meat (such as shoulder) was preferred over paler meat (such as loin).

The results of various studies have shown that many QTLs related to the most important breeding traits of pigs are located in SSC13. These include, among others, average daily gain, average backfat thickness, intramuscular fat content, loin lean-meat weight, daily feed intake, and feed conversion ratio during growth [49,50,51].

The SNP rs80860411 analyzed in the presented study was indicated as the main QTL in SSC13 for drip loss measured on the semimembranosus muscle. Integrated GWAS, eQTL, and QTT analysis was performed to identify candidate genes for pork meat quality. The rs80860411 SNP was the peak cis-eQTL SNP for the *GALNT15* gene. Additionally, it was shown that the expression level of *GALNT15* was significantly negatively correlated with the drip loss trait. Thus, *GALNT15* was proposed as a strong candidate gene affecting drip loss [29].

The *GLANT15* (also known as *GALNTL2*) gene is localized on porcine chromosome 13 (NCBI, Gene ID: 100157572, updated on 17 August 2024). The gene product catalyzes the initial reaction in O-linked oligosaccharide biosynthesis (the transfer of an N-acetyl-D-galactosamine residue to a serine or threonine residue on the protein receptor). It is able to transfer GalNAc residues to the Muc5AC peptide [52]. Protein glycosylation is an essential process in all eukaryotes. As reviewed by Varki (2017) [53], a wide variety of types of protein glycosylation occur in animals, plants, and microorganisms. It plays a significant role in the process of protein folding, their transport to target sites, as well as their activity and localization within the cell. In the body, glycoproteins participate in many biological processes, and their most important functions include cell recognition, differentiation, and development. They also participate in signal transduction [54]. O-GlcNAc glycosylation helps maintain skeletal muscle structure and function. As a result of glycosylation, the conformational stability and solubility of proteins, as well as the thermal stability of enzymes, are significantly increased [55]. Muscle proteome analysis revealed the presence of many O-GlcNAc-modified proteins [56], and among them, several key contractile proteins of striated muscles were identified [57]. A specific role of O-GlcNAcylation in acute skeletal muscle damage has also been demonstrated. It may become an important issue in the field of non-hereditary muscle diseases [55].

It has been described that due to the increasing incidence of obesity, insulin resistance, and hyperlipidemia, the number of patients with fatty liver disease is still increasing. Thanks to multidisciplinary studies, a list of 208 genes co-expressed in fibroblasts from two patients with fatty liver disease was obtained. Among them was the *GALNTL2* gene [58].

In the study by Funari et al. [59], 161 cartilage-selective genes were identified in humans, including *GALNTL2*. These genes have been identified as highly expressed in cartilage, but with low expression and low variability in 34 non-cartilage tissues.

Valdés-Hernández et al. [60] identified potential target genes related to FA (fatty acids) metabolism, which play a role in lipid metabolism (e.g., *ADIPOQ*, *FGFR4*, *PLIN1*, *NEU3*, *TFRC*), carbohydrate metabolism (e.g., *GALNT15*, *ADIPOQ*, *NEU3*, *PPP1R3D*), glucose metabolism (*MAFA*), and ion binding (e.g., *ADIPOQ*, *GOT1*, *SOD3*, *NUDT14*). Among the listed FA-correlated genes, *GALNT15* is included in the group of genes involved in meat quality. The results of this experiment may facilitate the implementation of breeding strategies based on the use of functional information and deepen the knowledge of gene regulation in pig muscle [60]. The fattening process has a significant impact on the quality of meat and fattening performance. The leading role in meat quality is played by the level of intramuscular fat (IMF). It determines the taste, texture (e.g., tenderness and water holding capacity), and is also a flavor carrier [61]. Increasing the lean meat content negatively correlates with fattening traits [62]. Through rigorous selection techniques, the efficiency of lean meat production has been improved. On the other hand, this contributed to a decrease in the quality and nutritional value of pork [63].

The analysis of the expression of mRNA, miRNA, and lncRNA in porcine *M. longissimus lumborum* (Fat-Type and Lean-Type) revealed differential expression levels of many genes (including the one encoding the GALNT15 protein), as well as miRNAs and lncRNAs regulating their activity. It was found that GALNT15 played a positive role in lipid deposition [64]. Recently, Takahashi et al. [65] reported that the expression of *GALNT15* was increased during adipogenesis in the human preadipocyte cell line. Overexpression of *GALNT15* increased the mRNA levels of CCAAT-enhancer binding protein (C/EBPα) and leptin. Knockdown of *GALNT15* suppressed the mRNA expression of adipocyte marker genes with a reduction of lipid accumulation and a decrease of the percentage of cells with oil droplets [65].

The intergenic rs80860411A>C polymorphism is located relatively close to the *GALNT15* gene [66], and it may be important in regulating its expression. Many SNPs associated with human disease are located in intergenic regions. They are considered to have potential regulatory functions for DNA sequences. As a result, conformational changes in the DNA structure may occur by affecting the chromatin state and interactions between distant loci. Protein–DNA or RNA–DNA interactions may also be disrupted, which may change the binding of promoters and enhancers by such molecules as regulatory proteins or RNA [67]. To confirm the potential involvement of rs80860411 in the regulatory processes of the *GALNT15* gene, separate studies would be required in this area.

## 5. Conclusions

In this study, the association between the intergenic SNP (rs80860411) and fattening, slaughter, and quality traits in Polish pigs (Polish Large White and native Pulawska breed) was analyzed. Statistically significant differences were found between the genotypes and traits studied. In the case of the studied polymorphism in the Pulawska breed, selection improving simultaneously fattening traits and meat quality cannot be carried out. A preference towards the *AA* genotypes may contribute to worsening fattening traits (lower growth rate and higher feed intake). The *AA* genotype, only in the case of the Polish Large White breed, improved the quality of the ham meat texture, without worsening fattening and slaughter parameters.

Our studies can be considered as a first step towards determining the functionality of the rs80860411. In our opinion, the studied SNP has a real potential to be QTN for some fattening, slaughter, and quality traits in pigs, especially in the native Pulawska breed. The obtained results may be helpful for its future maintenance.

## Figures and Tables

**Table 1:** Primer sequences and restriction fragment lengths (*A/C*, rs80860411).

Forward Reverse	*5′-CCACCCCAGACCTCTTGAAT-3′* *5′-GACTCTAGACTGAAGGCCCC-3′*
Amplicon length (bp)	842
Restriction fragments (bp)	*AA*—474 and 368 *AC*—842, 474 and 368 *CC*—842 (Not digested)

bp—base pairs.

**Table 2:** Genotype and allele frequencies of rs80860411A>C SNP and calculation of Hardy–Weinberg equilibrium.

Breed	N	Genotype			Allele		HWE	
		*AA*	*AC*	*CC*	*A*	*C*	ꭓ^2^	*P*
Polish Large White	187	0.04 (*n* = 7)	0.42 (*n* = 79)	0.54 (*n* = 101)	0.25	0.75	3.1896	0.0741
Pulawska	48	0.12 (*n* = 6)	0.48 (*n* = 23)	0.40 (*n* = 19)	0.36	0.64	0.0561	0.8127

*n*—number of individuals, HWE—Hardy–Weinberg equilibrium.

**Table 3:** Association between gene polymorphism and fattening performance traits in the analyzed breeds (LSM ± SE, least squares mean ± standard error).

Trait	Genotype	Polish Large White	Pulawska	Whole Population
Number of days on test [days]	*AA*	79.354 ± 5.39	**100.333 ± 3.40 ^A^**	84.772 ± 1.75
	*AC*	83.738 ± 3.65	**85.217 ± 1.74 ^B^**	83.695 ± 1.41
	*CC*	82.628 ± 3.72	**88.105 ± 1.91 ^B^**	83.678 ± 2.35
Daily feed intake [kg]	*AA*	2.396 ± 0.15	2.138 ± 0.06	2.405 ± 0.04
	*AC*	2.319 ± 0.10	2.277 ± 0.03	2370 ± 0.03
	*CC*	2.365 ± 0.10	2.244 ± 0.03	2373 ± 0.06
Lifetime daily gain [g/day]	*AA*	592.653 ± 36.73	**498.833 ± 23.20 ^a^**	564.981 ± 9.95
	*AC*	566.227 ± 24.86	**569.130 ± 11.85 ^b^**	568.687 ± 7.99
	*CC*	574.518 ± 25.33	**535.684 ± 13.04 ^b^**	549.649 ± 14.49
Test daily gain [g/day]	*AA*	883.968 ± 58.63	**721.167 ± 27.90 ^Aa^**	853.293 ± 18.8
	*AC*	858.058 ± 39.69	**829.30 ± 14.25 ^B^**	857.247 ± 14.62
	*CC*	866.307 ± 40.44	**805.158 ± 15.64 ^b^**	854.655 ± 26.48
Feed conversion rate [kg/kg gain]	*AA*	2.708 ± 0.11	**2.988 ± 0.07 ^a^**	2.842 ± 0.03
	*AC*	2.710 ± 0.08	**2.750 ± 0.04 ^b^**	2.780 ± 0.03
	*CC*	2.737 ± 0.08	**2.798 ± 0.04 ^a^**	2.792 ± 0.05
Age at slaughter [days]	*AA*	169.980 ± 11.07	**204.833 ± 7.98 ^a^**	180.146 ± 3.43
	*AC*	180.444 ± 7.49	**178.304 ± 4.08 ^b^**	178.925 ± 2.52
	*CC*	176.930 ± 7.64	**189.105 ± 4.48 ^b^**	184.490 ± 4.56

Bold typed text indicates statistically significant difference: superscript indices ^a^, ^b^—values in the columns for a given trait with different letters differ significantly at *p* ≤ 0.05, superscript indices ^A^, ^B^—values in the columns for a given trait with different letters differ significantly at *p* ≤ 0.01.

**Table 4:** Association between rs80860411 polymorphism and slaughter traits, taking into account the right half-carcass, in the analyzed breeds (LSM ± SE, least squares mean ± standard error).

Trait	Genotype	Polish Large White	Pulawska	Whole Population
Carcass yield [%]	*AA*	75.973 ± 0.40	76.230 ± 0.30	76.798 ± 0.14
	*AC*	76.031 ± 0.27	76.420 ± 0.15	76.799 ± 0.11
	*CC*	76.097 ± 0.28	76.393 ± 0.17	76.814 ± 0.21
Middle length of carcass [cm]	*AA*	77.603 ± 1.04	78.625 ± 0.85	79.181 ± 0.77
	*AC*	78.764 ± 0.70	78.452 ± 0.44	79.563 ± 0.62
	*CC*	78.491 ± 0.71	78.203 ± 0.48	79.169 ± 1.12
Loin weight [kg]	*AA*	7.439 ± 0.27	7.272 ± 0.23	7.772 ± 0.11
	*AC*	7.327 ± 0.18	7.700 ± 0.12	7.721 ± 0.09
	*CC*	7.355 ± 0.19	7.545 ± 0.13	7.660 ± 0.16
Loin weight without skin and backfat [kg]	*AA*	5.882 ± 0.26	5.736 ± 0.20	6.093 ± 0.09
	*AC*	5.865 ± 0.17	6.079 ± 0.10	6.065 ± 0.08
	*CC*	5.855 ± 0.18	5.840 ± 0.11	5.899 ± 0.14
Ham weight without skin and backfat [kg]	*AA*	9.538 ± 0.26	9.184 ± 0.22	**9.213 ± 0.10 ^b^**
	*AC*	9.294 ± 0.17	9.214 ± 0.11	**9.234 ± 0.08 ^b^**
	*CC*	9.310 ± 0.18	8.823 ± 0.12	**8.859 ± 0.15 ^a^**
Loin eye area [cm^2^]	*AA*	53.631 ± 2.35	**52.217 ± 2.36 ^a^**	**51.379 ± 0.86 ^B^**
	*AC*	51.196 ± 1.58	**53.972 ± 1.20 ^a^**	**53.057 ± 0.69 ^B^**
	*CC*	51.518 ± 1.61	**49.587 ± 1.32 ^b^**	**48.675 ± 1.25 ^A^**
Width of loin eye [cm]	*AA*	10.188 ± 0.35	**10.431 ± 0.32 ^A^**	**10.373 ± 0.13 ^B^**
	*AC*	10.195 ± 0.23	**10.554 ± 0.16 ^A^**	**10.581 ± 0.10 ^B^**
	*CC*	10.295 ± 0.24	**9.778 ± 0.18 ^B^**	**9.790 ± 0.18 ^A^**
Height of loin eye [cm]	*AA*	6.741 ± 0.28	6.977 ± 0.25	6.919 ± 1.10
	*AC*	6.562 ± 0.18	6.924 ± 0.13	6.875 ± 0.03
	*CC*	6.608 ± 0.19	6.772 ± 0.14	6.718 ± 0.15
Average backfat thickness of five measurements [cm]	*AA*	1.123 ± 0.14	1.295 ± 0.13	1.395 ± 0.05
	*AC*	1.122 ± 0.09	1.371 ± 0.07	1.361 ± 0.04
	*CC*	1.177 ± 0.10	1.527 ± 0.07	1.538 ± 0.08
Carcass meat content [%]	*AA*	63.155 ± 1.35	**61.339 ± 1.18 ^A^**	**61.765 ± 0.51 ^B^**
	*AC*	61.939 ± 0.90	**62.276 ± 0.60 ^A^**	**61.986 ± 0.41 ^B^**
	*CC*	62.117 ± 0.92	**59.254 ± 0.66 ^B^**	**59.131 ± 0.74 ^A^**
Weight of primary cuts [kg]	*AA*	24.261 ± 0.53	**23.621 ± 0.45 ^A^**	**23.952 ± 0.20 ^b^**
	*AC*	23.797 ± 0.35	**23.993 ± 0.23 ^A^**	**24.044 ± 0.16 ^b^**
	*CC*	23.859 ± 0.36	**22.838 ± 0.25 ^B^**	**22.954 ± 0.29 ^A^**

Bold typed text indicates statistically significant difference: superscript indices ^a^, ^b^—values in the columns for a given trait with different letters differ significantly at *p* ≤ 0.05, superscript indices ^A^, ^B^—values in the columns for a given trait with different letters differ significantly at *p* ≤ 0.01.

**Table 5:** Association between rs80860411 polymorphism and meat quality traits (LSM ± SE, least squares mean ± standard error).

Trait	Genotype	Polish Large White	Pulawska	Whole Population
Intramuscular fat content [%]	*AA*	1.542 ± 0.09	1.184 ± 0.08	1.149 ± 0.03
	*AC*	1.180 ± 0.05	1.210 ± 0.04	1.200 ± 0.03
	*CC*	1.166 ± 0.05	1.190 ± 0.04	1.176 ± 0.05
Meat color— luminosity [L*]	*AA*	51.345 ± 1.25	53.463 ± 0.72	52.780 ± 0.36
	*AC*	51.692 ± 0.85	53.943 ± 0.37	52.567 ± 0.29
	*CC*	51.935 ± 0.86	54.600 ± 0.40	53.371 ± 0.52
Meat color— redness [a*]	*AA*	17.653 ± 0.97	**17.612 ± 0.40 ^a^**	18.306 ± 0.23
	*AC*	17.917 ± 0.66	**16.450 ± 0.20 ^b^**	18.213 ± 0.19
	*CC*	17.699 ± 0.67	**16.545 ± 0.22 ^b^**	18.086 ± 0.34
Meat color— yellowness [b*]	*AA*	4.135 ± 0.82	2.208 ± 0.25	5.149 ± 0.18
	*AC*	4.325 ± 0.58	2.297 ± 0.13	5.189 ± 0.14
	*CC*	4.381 ± 0.60	2.372 ± 0.14	5.273 ± 0.26
pH45 *M. longissimus lumborum*	*AA*	6.055 ± 0.10	6.338 ± 0.07	6.199 ± 0.04
	*AC*	6.090 ± 0.07	6.272 ± 0.03	6.215 ± 0.02
	*CC*	6.104 ± 0.07	6.319 ± 0.04	6.245 ± 0.05
pH24 *M. longissimus lumborum*	*AA*	5.557 ± 0.04	5.665 ± 0.03	5.601 ± 0.02
	*AC*	5.557 ± 0.05	5.637 ± 0.02	5.591 ± 0.01
	*CC*	5.569 ± 0.04	5.640 ± 0.02	5.590 ± 0.02
pH45 *M. semimembranosus*	*AA*	6.288 ± 0.09	6.243 ± 0.06	6.356 ± 0.03
	*AC*	6.358 ± 0.06	6.266 ± 0.03	6.345 ± 0.03
	*CC*	6.360 ± 0.06	6.283 ± 0.03	6.369 ± 0.48
pH24 *M. semimembranosus*	*AA*	5.685 ± 0.05	5.623 ± 0.06	5.676 ± 0.02
	*AC*	5.681 ± 0.03	5.632 ± 0.04	5.648 ± 0.02
	*CC*	5.692 ± 0.03	5.679 ± 0.03	5.702 ± 0.03
Cooking loss—loin (%)	*AA*	28.241 ± 1.46	27.080 ± 1.58	27.879 ± 0.72
	*AC*	28.163 ± 0.49	27.328 ± 0.84	27.868 ± 0.50
	*CC*	28.271 ± 0.42	27.877 ± 0.91	28.475 ± 1.15
Cooking loss—ham (%)	*AA*	32.720 ± 3.29	32.620 ± 1.60	33.441 ± 0.55
	*AC*	34.397 ± 0.99	32.927 ± 0.82	32.748 ± 0.37
	*CC*	33.301 ± 0.78	32.264 ± 0.93	32.292 ± 0.88
Water holding capacity	*AA*	24.532 ± 3.08	32.273 ± 2.83	25.577 ± 1.14
	*AC*	26.420 ± 2.09	33.995 ± 1.47	26.377 ± 0.92
	*CC*	26.394 ± 2.12	36.115 ± 1.60	28.687 ± 1.67

Bold typed text indicates statistically significant difference: superscript indices ^a^, ^b^—values in the columns for a given trait with different letters differ significantly at *p* ≤ 0.05.

**Table 6:** Association between rs80860411 polymorphism and the loin texture (LSM ± SE, least squares mean ± standard error).

Trait	Genotype	Polish Large White	Pulawska	Whole Population
Firmness (r)	*AA*	**18.534 ± 4.27 ^b^**	16.030 ± 4.10	24.025 ± 1.59
	*AC*	**21.113 ± 1.42 ^b^**	19.210 ± 2.19	22.625 ± 1.11
	*CC*	**25.893 ± 1.23 ^a^**	23.628 ± 2.37	28.061 ± 2.57
Toughness (r)	*AA*	56.779 ± 12.73	53.800 ± 12.47	64.987 ± 4.71
	*AC*	59.880 ± 4.24	50.448 ± 6.67	62.226 ± 3.27
	*CC*	69.097 ± 3.65	70.088 ± 7.20	81.735 ± 7.57
Firmness	*AA*	63.713 ± 8.38	63.498 ± 7.18	76.140 ± 3.32
	*AC*	79.516 ± 2.79	78.843 ± 3.84	79.691 ± 2.31
	*CC*	78.498 ± 2.40	72.262 ± 4.15	75.730 ± 5.34
Toughness	*AA*	161.377 ± 21.77	146.483 ± 18.11	179.135 ± 9.09
	*AC*	187.019 ± 7.26	192.796 ± 9.68	193.171 ± 6.32
	*CC*	185.649 ± 6.25	166.577 ± 10.46	174.124 ± 14.63
Hardness	*AA*	5.665 ± 1.64	6.796 ± 1.42	7.243 ± 0.66
	*AC*	7.046 ± 0.55	7.168 ± 0.76	7.943 ± 0.46
	*CC*	7.430 ± 0.47	7.369 ± 0.82	8.072 ± 1.06
Springiness	*AA*	0.714 ± 0.03	0.686 ± 0.02	0.696 ± 0.01
	*AC*	0.684 ± 0.01	0.686 ± 0.01	0.694 ± 0.01
	*CC*	0.686 ± 0.01	0.671 ± 0.01	0.680 ± 0.02
Cohesiveness	*AA*	0.655 ± 0.02	0.637 ± 0.02	0.634 ± 0.01
	*AC*	0.623 ± 0.01	0.631 ± 0.01	0.638 ± 0.01
	*CC*	0.623 ± 0.01	0.612 ± 0.01	0.616 ± 0.02
Chewiness	*AA*	2.614 ± 0.82	3.081 ± 0.71	3.334 ± 0.34
	*AC*	3.380 ± 0.27	3.196 ± 0.38	3.652 ± 0.24
	*CC*	3.393 ± 0.24	3.237 ± 0.41	3.648 ± 0.55
Resilience	*AA*	0.278 ± 0.01	0.274 ± 0.01	0.272 ± 0.01
	*AC*	0.266 ± 0.01	0.271 ± 0.01	0.275 ± 0.01
	*CC*	0.267 ± 0.01	0.259 ± 0.01	0.262 ± 0.01

r = raw tissue, bold typed text indicates statistically significant difference: superscript indices ^a^, ^b^—values in the columns for a given traits with different letters differ significantly at *p* ≤ 0.05.

## Data Availability

The original contributions presented in this study are included in the article/Supplementary Material. Further inquiries can be directed to the corresponding author.

## Supplementary Materials

The following supporting information can be downloaded at https://www.mdpi.com/article/10.3390/ani15142090/s1.
