# Supplementary material for: The Effect of rs80860411 Polymorphism on Fattening, Slaughter, and Pork Quality Traits in Polish Large White and Pulawska Breeds
Source: Animals (Basel). 2025 Jul 15;15(14):2090. doi: 10.3390/ani15142090 (PMC12291728; doi:10.3390/ani15142090)
Supplement: Supplementary file 1 [file animals-15-02090-s001.zip › animals-3762420-supplementary.pdf]

Table S1. Association between rs80860411 polymorphism and the loin texture taking into account the slaughter date (LSM±SE, least squares mean ± standard error).

Trait	Genotype	Polish Large White	Pulawska	Whole Population
Firmness (r)	*AA*	21.656 ± 4.92	14.879 ± 3.97	30.776 ± 2.28
	AC	25.263 ± 2.67	19.046 ± 2.10	28.767 ± 1.94
	CC	29.646 ± 2.56	21.327 ± 2.51	33.983 ± 2.96
Toughness (r)	*AA*	66.843 ± 14.57	51.175 ± 12.37	81.018 ± 6.84
	AC	74.358 ± 7.89	50.073 ± 6.56	77.003 ± 5.82
	CC	82.028 ± 7.59	64.837 ± 7.82	95.038 ± 8.88
Firmness	*AA*	69.971 ± 9.69	64.452 ± 7.25	83.248 ± 4.88
	AC	85.625 ± 5.25	78.980 ± 3.84	86.691 ± 4.16
	CC	83.423 ± 5.05	74.169 ± 4.58	83.032 ± 6.34
Toughness	*AA*	178.811 ± 25.05	146.888 ± 18.49	196.838 ± 13.35
	AC	206.114 ± 13.58	192.854 ± 9.80	210.836 ± 11.36
	CC	201.424 ± 13.05	167.387 ± 11.69	190.903 ± 17.33
Hardness	*AA*	6.287 ± 1.90	6.699 ± 1.45	10.634 ± 0.94
	AC	8.869 ± 1.03	7.154 ± 0.77	10.961 ± 0.79
	CC	8.529 ± 0.99	7.177 ± 0.92	11.152 ± 1.21
Springiness	*AA*	0.722 ± 0.03	0.687 ± 0.02	0.726 ± 0.02
	AC	0.690 ± 0.02	0.686 ± 0.01	0.715 ± 0.01
	CC	0.696 ± 0.02	0.673 ± 0.01	0.702 ± 0.02
Cohesiveness	*AA*	0.673 ± 0.03	0.635 ± 0.02	0.669 ± 0.01
	AC	0.638 ± 0.02	0.631 ± 0.01	0.663 ± 0.01
	CC	0.640 ± 0.01	0.609 ± 0.01	0.644 ± 0.02
Chewiness	*AA*	3.095 ± 0.95	3.059 ± 0.72	5.373 ± 0.46
	AC	4.075 ± 0.52	3.193 ± 0.38	5.362 ± 0.39
	CC	4.061 ± 0.50	3.195 ± 0.46	5.349 ± 0.60
Resilience	*AA*	0.289 ± 0.02	0.272 ± 0.01	0.293 ± 0.01
	AC	0.274 ± 0.01	0.271 ± 0.01	0.291 ± 0.01
	CC	0.276 ± 0.01	0.256 ± 0.01	0.280 ± 0.01

r = raw tissue.

Table S2. Association between rs80860411 polymorphism and the ham texture (LSM±SE, least squares mean ± standard error).

Trait	Genotype	Polish Large White	Pulawska	Whole Population
Firmness (r)	*AA*	18.318 ± 3.17	24.008 ± 2.03	24.433 ± 1.09
	AC	24.342 ± 0.95	24.190 ± 1.04	25.053 ± 0.70
	CC	24.563 ± 0.75	22.693 ± 1.17	23.466 ± 1.66
Toughness (r)	*AA*	50.096 ± 11.58	72.063 ± 6.85	72.861 ± 3.61
	AC	73.912 ± 3.49	67.500 ± 3.50	72.295 ± 2.47
	CC	76.073 ± 2.75	67.312 ± 3.95	71.281 ± 5.83

 https://doi.org/10.3390/xxxxx

Firmness	*AA*	78.068 ± 9.47	92.502 ± 8.70	86.394 ± 3.77
	AC	88.471 ± 2.81	77.666 ± 4.45	84.586 ± 2.58
	CC	83.655 ± 2.24	89.876 ± 5.03	94.100 ± 6.09
Toughness	*AA*	187.639 ± 22.52	211.033 ± 19.17	208.873 ± 9.66
	AC	214.224 ± 6.79	190.338 ± 9.79	205.890 ± 6.60
	CC	203.562 ± 5.34	210.536 ± 11.07	222.421 ± 15.58
Hardness	*AA*	9.177 ± 8.81	10.802 ± 1.58	11.767 ± 0.70
	AC	15.398 ± 2.66	10.641 ± 0.81	10.595 ± 0.48
	CC	10.715 ± 2.09	8.596 ± 0.91	8.759 ± 1.13
Springiness	*AA*	0.724 ± 0.04	0.659 ± 0.03	0.713 ± 0.02
	AC	0.713 ± 0.01	0.726 ± 0.02	0.709 ± 0.01
	CC	0.721 ± 0.01	0.713 ± 0.02	0.710 ± 0.03
Cohesiveness	*AA*	0.642 ± 0.03	0.575 ± 0.03	0.593 ± 0.07
	AC	0.611 ± 0.01	0.632 ± 0.02	0.662 ± 0.05
	CC	0.615 ± 0.01	0.625 ± 0.02	0.652 ± 0.11
Chewiness	*AA*	4.576 ± 1.15	4.362 ± 0.87	5.252 ± 0.37
	AC	4.539 ± 0.35	4.958 ± 0.45	4.901 ± 0.25
	CC	5.012 ± 0.27	3.965 ± 0.50	4.103 ± 0.59
Resilience	*AA*	0.274 ± 0.02	0.231 ± 0.02	0.251 ± 0.01
	AC	0.249 ± 0.01	0.261 ± 0.01	0.264 ± 0.01
	CC	0.256 ± 0.01	0.253 ± 0.01	0.259 ± 0.01

r = raw tissue.

Table S3. Association between rs80860411 polymorphism and the ham texture taking into account the slaughter date (LSM±SE, least squares mean ± standard error).

Trait	Genotype	Polish Large White	Pulawska	Whole Population
Firmness (r)	*AA*	**17.054 ± 3.40**[a]	23.711 ± 2.04	25.489 ± 1.15
	AC	**25.323 ± 1.58**[b]	24.229 ± 1.03	25.725 ± 1.38
	CC	**25.287 ± 1.38**[b]	22.197 ± 1.24	23.818 ± 1.95
Toughness (r)	*AA*	**49.942 ± 12.38**[a]	70.738 ± 6.80	71.944 ± 5.40
	AC	**81.168 ± 5.75**[b]	67.672 ± 3.45	70.790 ± 4.94
	CC	**81.420 ± 5.04**[b]	65.104 ± 4.15	67.853 ± 6.98
Firmness	*AA*	77.414 ± 10.39	93.308 ± 8.82	88.775 ± 6.13
	AC	88.622 ± 4.83	77.560 ± 4.47	87.320 ± 5.61
	CC	83.202 ± 4.23	91.220 ± 5.39	96.364 ± 7.93
Toughness	*AA*	202.238 ± 24.55	213.065 ± 19.40	207.530 ± 15.73
	AC	232.993 ± 11.39	190.073 ± 9.83	204.252 ± 14.40
	CC	220.631 ± 9.99	213.922 ± 11.84	219.977 ± 20.34
Hardness	*AA*	9.306 ± 9.56	10.931 ± 1.61	11.500 ± 1.08
	AC	17.106 ± 4.44	10.624 ± 0.81	10.968 ± 0.99
	CC	11.411 ± 3.89	8.811 ± 0.58	9.416 ± 1.40
Springiness	*AA*	0.738 ± 0.04	0.664 ± 0.03	0.735 ± 0.02
	AC	0.728 ± 0.02	0.726 ± 0.02	0.734 ± 0.02
	CC	0.733 ± 0.02	0.722 ± 0.02	0.748 ± 0.03
Cohesiveness	*AA*	0.641 ± 0.03	0.573 ± 0.03	0.679 ± 0.11

	AC	0.625 ± 0.01	0.633 ± 0.02	0.702 ± 0.10
	CC	0.628 ± 0.01	0.621 ± 0.02	0.703 ± 0.14
Chewiness	*AA*	4.682 ± 1.27	4.431 ± 0.89	5.723 ± 0.56
	AC	4.359 ± 0.59	4.949 ± 0.45	5.662 ± 0.51
	CC	4.832 ± 0.52	4.081 ± 0.54	5.108 ± 0.73
Resilience	*AA*	0.274 ± 0.02	0.230 ± 0.02	0.277 ± 0.01
	AC	0.257 ± 0.01	0.261 ± 0.01	0.283 ± 0.01
	CC	0.264 ± 0.01	0.252 ± 0.01	0.281 ± 0.01

r = raw tissue, bold typed text indicates statistically significant difference: superscript indices a, b – values in the columns for a given traits with different letters differ significantly at p≤0.05.
